# 3D Finite Element Simulation of Micro End-Milling by Considering the Effect of Tool Run-Out

**DOI:** 10.3390/mi8060187

**Published:** 2017-06-16

**Authors:** Ali Davoudinejad, Guido Tosello, Paolo Parenti, Massimiliano Annoni

**Affiliations:** 1 Department of Mechanical Engineering, Technical University of Denmark, Building 427A, Produktionstorvet, 2800 Kgs. Lyngby, Denmark; guto@mek.dtu.dk; 2 Mechanical Engineering Department, Politecnico di Milano, Via La Masa 1, 20156 Milan, Italy; paolo.parenti@polimi.it (P.P.); massimiliano.annoni@polimi.it (M.A.)

**Keywords:** micro milling, finite element, run-out, chip formation, cutting force, cutting temperature, 3D simulation, measurement

## Abstract

Understanding the micro milling phenomena involved in the process is critical and difficult through physical experiments. This study presents a 3D finite element modeling (3D FEM) approach for the micro end-milling process on Al6082-T6. The proposed model employs a Lagrangian explicit finite element formulation to perform coupled thermo-mechanical transient analyses. FE simulations were performed at different cutting conditions to obtain realistic numerical predictions of chip formation, temperature distribution, and cutting forces by considering the effect of tool run-out in the model. The radial run-out is a significant issue in micro milling processes and influences the cutting stability due to chip load and force variations. The Johnson–Cook (JC) material constitutive model was applied and its constants were determined by an inverse method based on the experimental cutting forces acquired during the micro end-milling tests. The FE model prediction capability was validated by comparing the numerical model results with experimental tests. The maximum tool temperature was predicted in a different angular position of the cutter which is difficult or impossible to obtain in experiments. The predicted results of the model, involving the run-out influence, showed a good correlation with experimental chip formation and the signal shape of cutting forces.

## 1. Introduction

Micro milling is one the prevalent micro manufacturing processes in terms of a high volume and low production cost in comparison to other processes to achieve high-precision three dimensional (3D) products. Micro milling is characterized by the mechanical interaction of a sharp tool with the workpiece material, causing fractures inside the material along defined paths, eventually leading to the removal of the workpiece material in the form of chips [1]. Micro machining presents numerous challenges that have been attracting the attention of both scientific and industrial research for the past three decades [2,3]. Machinability studies are mostly based on physical experimental testing and are used as supplementary validation for the modeling approach. In micro machining, due to the process downscaling, experimental testing has to tackle several complexities such as thermal transient, tool wear, tool run-out, fixture, and workpiece, etc. [3,4,5]. In micro milling, some undesired phenomena such as tool wear/breakage, surface location errors, burr formation, and chatter are highly dependent on the cutting process itself. The cutter run-out also influences the process. Consequently, the prediction capability and accuracy of models is critical in simulations. Even a few microns of cutter run-out can significantly affect the accuracy of micro end-milling and create extensive force variations as opposed to conventional milling [6,7].

During the last four decades, numerical modeling (Finite Element Modeling (FEM) methods) has been recognized as a viable method for reducing or eliminating trial and error approaches in the design and optimization of machining processes. The 2D FE model investigated the effects of three microgroove parameters (groove width, edge distance, and depth ratio) on the friction and wear of textured and non-textured tools. It was revealed that microgrooves on the rake face of a cutting tool perpendicular to the chip flow direction were effective in a way that the frictional behavior at the chip–tool interface was improved. Microgrooves were also very effective in reducing flank wear and crater wear [8].

This study reported a significant effect of temperature on the flow stress that affected the cutting forces prediction results. A strain gradient plasticity based-FE model of orthogonal micro cutting of Al5083-H116 was used to investigate the influence of the tool edge radius on the size effect [9]. A micro orthogonal cutting simulation at small uncut chip thickness levels was presented to predict a sizeable strain gradient strengthening effect that was validated through micro cutting experiments [10]. Irfan et al. investigated the 2D FE modeling of Inconel 718 micro milling for different feed rates and cutting tool edge angles. A higher cutting temperature was observed at a higher feed rate and with a negative edge angle. The maximum stress distribution occurred in the contact zone between the tool and chip [11].

Afazov et al. [12] investigated micro milling force calculations using orthogonal FE methods including a mathematical model for determining the uncut chip thickness in the presence of run-out effects for AISI 4340 steel. The uncut chip thickness and the tool path trajectories were determined for distinct micro milling parameters such as the cutting velocity, cutting tool radius, feed rate, and number of teeth. The prediction of 2D FEM orthogonal micro cutting forces by considering the chip load and tool edge radius effect was utilized for the micro end milling of brass 260 in [13]. Tool trajectories, the edge radius, and run-out effects are considered in the prediction of milling forces. The cutting force coefficients were identified from a set of simulations at a range of cutting edge radii and chip loads. Another study investigated coated and uncoated cubic boron nitride (CBN) tools with the 2D finite element method (FEM) to predict the chip formation, cutting forces, temperatures, and wear rates generated in the micro milling of Ti-6Al-4V titanium alloy. The effect of run-out was not considered in the model [14]. 2D FEM micro-end milling of Ti-6Al-4V titanium alloy was investigated with a plane strain-based orthogonal cutting force model with a tool edge radius effect to validate the cutting force results [15].

Although 2D FE simulations offer some distinct advantages when studying the orthogonal cutting process, 3D FE models provide more realistic oblique configurations, mainly in milling processes with complex cutting tool geometries [16,17]. 3D FE simulations present supplementary analysis capabilities to investigate the effect of the helix angle and tool edge radius on chip flow and burr formation, which are almost impossible to be considered by 2D FE models [18].

A 3D FE model was developed to investigate the correlation between the cutting temperature and cutting edge radii in the micro-end milling of Al2024-T6 [19]. In this study, the simulation results revealed that increasing the tool edge radius increases the cutting temperature, reaching the highest temperature of 57.5 °C at the edge radius. Another study investigated 3D FEM to predict tool and workpiece temperature fields in the micro milling process of Ti-6Al-4V under various cutting conditions. A thermocouple was used for the experimental temperature measurements to validate the simulation results [20]. A 3D FEM study was developed on the same Ti-6Al-4V material in order to investigate the cutting forces in three directions under different cutting conditions, and the chip evolution and morphologies of different cutting parameters were also analyzed. The predicted and experimental chip morphologies were compared and a good agreement was observed. In terms of cutting forces, the predicted cutting forces showed a good correlation with the experimentally measured data with a 6–8% prediction error in the *X*- and *Y*-directions and a 12–15% prediction error in the *Z*-direction [21].

A study was conducted using 3D FEM simulations to evaluate the cutting force predictions for different cutting conditions in the micro-end milling of Al6061-T6 and the results were compared against the experiments presented in [22]. 3D FE simulations were utilized to predict the chip flow and shape during micro end-milling of Ti-6Al-4V titanium alloy in [23]. The model was developed for studying the tool wear along the micro tool and investigating the influence of the cutting edge roundness increment on the machining process performance.

Even though many studies on micro end milling have demonstrated the capability to provide information on chip formation, cutting forces, tool stresses, and temperature distribution, very limited work in the scientific literature deals with FE simulation by considering the effect of tool run-out in an integrated FE model. In the aforementioned studies, run-out was considered by combining mathematical models or other methods to the orthogonal FE model in the case of complex micro end milling operations [12,13]. However, in this study, run-out was considered in an integrated model to carry out the simulation.

In order to better understand the cutting mechanism and the effects of run-out in micro milling, 3D simulations are required, but a very limited number of studies in the literature deal with micro milling 3D simulations. However, at a macro scale, a few examples are available [16,17,21,24] that can be used as a starting point to develop a 3D micro milling model. In the literature, there is still a significant gap in our understanding of the effect of run-out in micro milling with 3D integrated finite element modeling techniques validated with accurate experimental campaigns.

**Figure 1:**
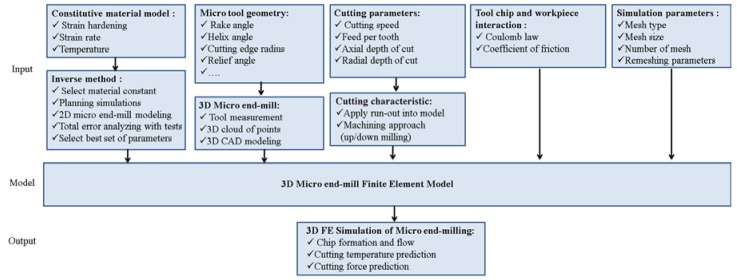
Flowchart for 3D micro end-milling temperature and force prediction.

The research presented in the current paper includes a 3D FE model of the micro end-milling process applied to different cutting parameters considering the effect of tool run-out and the tool edge radius for predicting the chip formation temperature distribution and cutting forces. The FE model flow chart is presented in Figure 1.

The paper is structured as follows. Section 2 introduces the FE modeling and methodology, where experimental cutting forces are used to define the material model constants through an inverse method. The FE model predictions are compared against a series of micro milling experiments and the procedures are detailed in Section 3. Finally, the chip formation temperature distribution and cutting forces are discussed in Section 4. Conclusions close the paper in Section 5.

## 2. 3D Finite Element Simulation Setup

When dealing with FE modeling, an adequate software selection is fundamental for obtaining reliable results [25]. The 3D model for micro end-milling Al6082-T6 alloy was simulated using an explicit time integration method by employing a Lagrangian finite element formulation to perform coupled thermo-mechanical transient analysis. The AdvantEdge^®^ FEM software (Version 6.2.011, Third Wave Systems, Minneapolis, MN, USA) was used to implement the FE model.

### 2.1. Tool Geometry and Modeling

**Figure 2:**
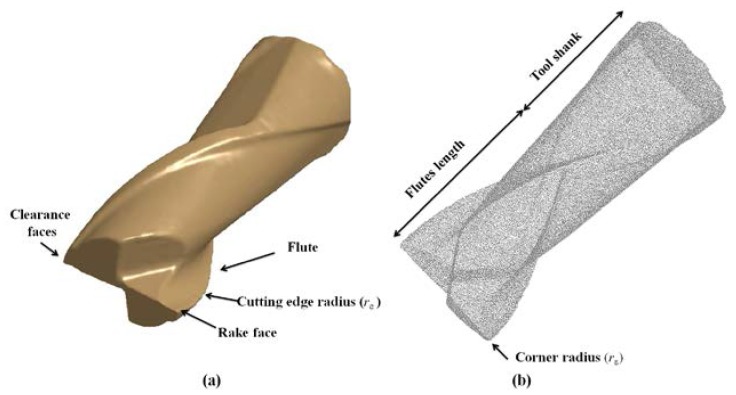
(**a**) Solid CAD model, (**b**) preliminary cloud of points.

The influence of the tool geometry on the chip formation, cutting temperature, tool wear, etc., was highlighted in the literature [24,26]. In this study, the selected cutting tool was a Dormer S150.05 micro flat end-mill with TiAlN-X (Titanium Aluminium Nitride Extreme) (Dormer, Sheffield, England) surface treatment. Table 1 shows the details of the nominal and actual characteristics of the tool geometry. The tools were inspected and measured by a 3D optical measuring system (Alicona Infinite Focus©) prior to machining (measurement parameters: 10× magnification, exposure time = 1.206 ms, contrast = 1, coaxial light, estimated vertical and lateral resolutions = 0.083 μm and 4 μm, respectively). The complex geometry of micro end-mills requires the use of a computer-aided design (CAD) model to obtain a complete and detailed representation of the cutter. The cutter’s initial cloud of points was acquired by the Alicona Infinite Focus© system and used to precisely describe the tool geometry, taking advantage of the high density of the acquired three dimensional clouds of points. Cleaning and filtering post-processing operations after the scanning acquisition were applied to generate a reconstructed CAD model suitable for the FEM. Figure 2 shows the CAD model and cloud of points of the simulated micro end-mill.

Details of the actual geometry of the cutter such as the corner edge radius, helix angle, etc. (Table 1), were measured and the values extracted to reconstruct the model. The 3D STereoLithography (STL) file format was used for the FEM analysis. The tool geometry is critical to obtain reliable responses from the model. Based on the preliminary analyses [27], the nominal tool geometry was not able to reproduce the experimental force measurements.

### 2.2. Cutting Configuration Setup

**Figure 3:**
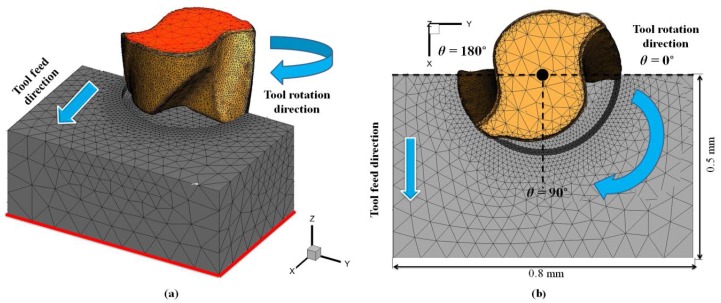
(**a**) 3D FEM setup perspective view, boundary condition (red area), (**b**) definition of tool engagement angle *θ* (top view).

Figure 3 shows the setup, boundary conditions, and general geometry of the 3D FEM simulation of full slot micro end-milling. The workpiece boundary nodes were fixed in the *XY* and *Z* bottom directions and the tool constrained in the *Z* top direction, as shown in red in Figure 3a; the feed was applied by moving the tool along the *X* direction. The tool and workpiece were kept at ideal dimensions to maintain steady state cutting conditions and a minimum simulation time. The cutting tool was considered as a rigid body and the workpiece was considered as a viscoplastic material. The tool and workpiece were meshed with four node tetrahedral elements, for a total number of 56,648 and 60,916 elements, and 14,785 and 11,500 nodes, respectively, for full slot milling.

After preliminary evaluation tests [28,29], the initial meshing parameters of the workpiece were set as 2 mm and 0.001 mm in terms of the maximum and minimum element size, respectively. The maximum element size of the cutter was set at 1 mm and the minimum at 0.001 mm. Other meshing parameters such as mesh grading = 0.5 (to determine the nature of transition), curvature safety = 3 (to determine the mesh accuracy), and segments per edge = 1 (to determine the density of nodes on the unit length of any edge) were selected. The choice of other meshing parameters was coherent with the recent study on meshing strategies in an FEM simulation [30]. The proper selection of meshing parameters is of great importance since it affects the accuracy of the simulation results and the computation time. A higher mesh density was considered in the area near the cutting zone in order to increase the accuracy of the computed outputs.

Adaptive remeshing was applied in order to avoid the inaccuracies due to elemental distortion, inherent to the Lagragian formulation. The mesh quality was constantly monitored during the simulations and when the element distortion reached a certain tolerance, adaptive remeshing was triggered. In addition, refinement and coarsening operators were applied in various parts of the mesh. The mesh was refined where the plastic deformation was active and coarsened in inactive regions [26].

The simulations were performed on a computer equipped with a processor characterized by 2.6 GHz, 16 cores, and 64 GB RAM. An eight threads parallel simulation mode was used to speed up the calculation time about six times. The 3D FE simulation time for a full rotation (360°) was about 25 h.

### 2.3. Radial Run-Out

Due to the significant effect of run-out in micro end-milling [6,7], the radial run-out (i.e., eccentricity) of the cutting tool was considered in the FE model. In the first approximation, the essential source of run-out is the cutting tool center offset [12]. The run-out definition from the ASME B89.3.4-2010 standard (“Axes of Rotation: Methods for Specifying and Testing”) is “the total displacement measured by an indicator sensing against a moving surface or moved with respect to a fixed surface” [31]. This definition, applied to the case of tool run-out in milling, introduces the run-out as composed by the “spindle error motion”, the tool “roundness error” (difference among cutting edge radii), and the tool-spindle “centering errors”. The spindle error motion is defined as the incorrect motion of the spindle rotating axis around a reference axis; sources of error motion are bearings inaccuracies and/or the system dynamics [31].

**Figure 4:**
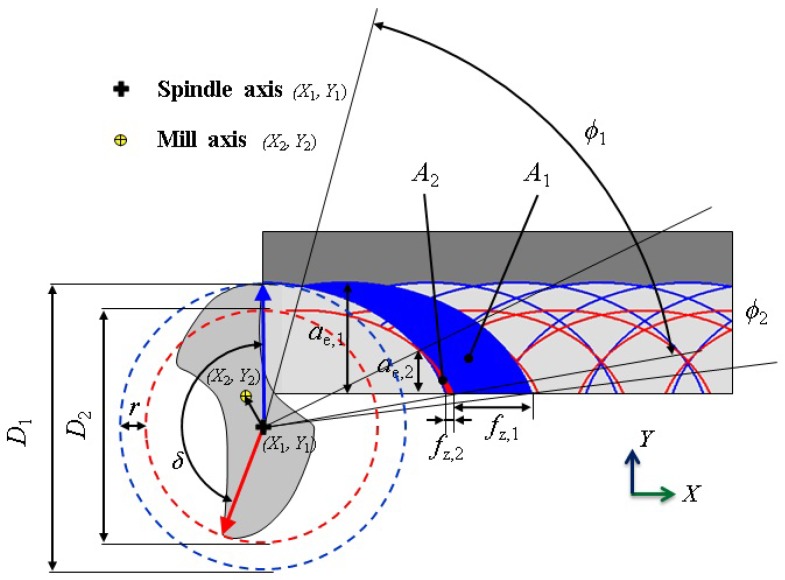
Definitions and cutting edge trajectories for a radial run-out affected micro end-mill.

Figure 4 represents a two-flute micro mill affected by run-out involving a centering error, indicated by the arrow between the spindle and the mill axes, and a roundness error. “Cutting edge 1”, the most engaged cutting edge describing the big dashed blue circle (*D*_1_), is affected by the roundness error that makes its radius shorter than the nominal value. The centering error and roundness error partially compensate for this in Figure 4. During the mill rotation around the spindle axis, the less engaged cutting edge, named “cutting edge 2”, describes the small dashed red circle (*D*_2_). Tool radial run-out makes the cutting edge trajectories different from the ideal identical cycloids (Figure 4); this fact makes the chip thickness produced by one cutting edge different from the other one. Consequently, the effective engagement angles (*φ*_1_ and *φ*_2_), the effective removed areas (*A*_1_ and *A*_2_), the effective widths of cut (*a_e_*_,1_ and *a_e_*_,2_), and the feeds per tooth (*f_z_*_,1_ and *f_z_*_,2_) of the two cutting edges are different in the engagements, depending on the mill centering and roundness errors.

In the developed 3D FE model, the radial run-out was considered and the center of the tool was shifted by 3 μm, corresponding to the experimental run-out measurement, along the *X* and *Y* directions of the Cartesian coordinate system. Such deviations were measured by the visual tool setter (VTS©) (Marposs, Bologna, Italy) installed on the machine (see Section 3 for details of the experimental setup).

### 2.4. Contact Friction Modeling

The friction phenomenon at the chip-tool interface was modeled using the Coulomb friction, as shown in Equation (1).

(1) $$\tau =\mu \sigma_{n}$$

The frictional stresses τ on the tool rake face are assumed to be proportional to the normal stresses $\sigma_{n}$ with a coefficient of friction μ. According to Özel [32], the sliding friction can be dominant during low cutting speed machining and the sticking friction is dominant during high-speed machining. In micro milling, even if high rotational speeds are used, the cutting speed is lower (in this study about 31 m/min) than in macro milling due to the small tool diameter. Consequently, the Coulomb sliding friction model can be considered as effective for the micro milling process. The constant value of the friction coefficient was *μ* = 0.7 in this study. This value was selected based on the experimental identification by Medaska on Al6061-T6 with carbide tools [33]. Al6082-T6 and Al6061-T6 are two popular aluminum alloys and sometimes they replace each other in the industrial practice due to similar characteristics.

### 2.5. Constitutive Material Model

The reliability of the finite element model results are greatly influenced by the material constitutive law and contact conditions at tool-chip and tool-workpiece interfaces [34]. A reliable material model that perfectly captures the constitutive behavior of the alloy under high strain, strain rate, and temperature is critical in machining simulations. A number of different constitutive material models have been reported in the literature such as Oxley’s constitutive model [35], the Power law model [26], the strain path dependent model [36], and the Johnson-Cook (JC) model [37], etc. Among the different constitutive material models, JC is widely used for machining simulations due to the corresponding material behavior as a function of the strain, strain rate, and temperature [38]. In the present study, the micro end-mill is modelled as a rigid body for the coated carbide tool (TiAlN-X coating with 2 μm thickness) with the Advantedge tool default material. However, the workpiece is considered as viscoplastic material and an essential input is the accurate definition of the workpiece material properties and the constitutive material model is represented by the Johnson-Cook model. The flow stress can be expressed as (Equation (2)):

(2) $$\sigma =\left(A+B\;{\left(\varepsilon \right)}^{n}\right)\left[1+C\ln\left(\frac{\varepsilon^{\cdot }}{{\varepsilon^{\cdot }}_{0}}\right)\right]\left[1-{\left(\frac{T-T_{a}}{T_{m}-T_{a}}\right)}^{m}\right]$$

where σ is the material flow stress, ε is the plastic strain, $\varepsilon^{\cdot }$ is the strain rate, and ${\varepsilon^{\cdot }}_{0}$ is the reference strain rate. T is the material temperature, $T_{m}$ is the melting point, and $T_{a}$ is the room temperature. The JC constants are as follows: *A* is the yield stress, *B* is the pre-exponential factor, *C* is the strain rate factor, *n* is the work hardening exponent, and *m* is the thermal softening exponent.

#### Inverse JC Parameters Estimation with 2D FE Simulations

The correct calibration of these parameters is critical to predict the forces, temperature, chip morphology, etc., with a reasonable accuracy. Unfortunately, there is a lack of standardization for these coefficients in the literature. The discrepancies are due to the different methods and test conditions used for the determination of the material constants [39]. To reduce the uncertainty associated with the parameters value determination, a viable method based on a machining test was adopted in this paper. It consists of an inverse method, as used by a number of researchers to obtain the material constants [39,40,41]. In particular, an experimental procedure for identifying the material constants at different cutting conditions was developed. An experimental campaign was specifically carried out. Table 2 shows its cutting conditions and the maximum experimental cutting force measured values. The force measuring system and compensation method applied for this set of experiments and for the final validation tests are detailed in the experimental procedure (Section 3). Each experiment was replicated two times to consider the experimental variability. Four parameters of the Johnson-Cook equation (*A*, *B*, *n*, *C*), which were the most susceptible to the strain hardening effect, were selected.

The simulations plan was based on a 2^4^ full factorial design (two levels and 4 factors, Table 3). 16 JC parameter combinations were used for simulating each cutting condition of Table 2, for a total of 64 runs. An initial JC parameters combination for the 6082-T6 aluminum alloy was taken from (Table 3). These values were obtained at high strain rates by conducting split Hopkinson pressure bar (SHPB) tests in conditions similar to metal cutting. The other level was obtained by scaling down the [42] values by 50%, apart from the *m* one (Table 3). This scale was applied due to the overestimated result of the cutting force simulations with original values [27]. Other physical properties of Al6082-T6 used in the FE model are presented in Table 4 [43].

The FEM simulations in the inverse method were carried out to evaluate the best combination of JC parameters for obtaining the most accurate cutting forces. A simplified 2D FE model was used to reduce the computational effort to carry out the total 64 simulations, under the hypothesis that the best JC parameters would also perform well in the 3D case.

**Figure 5:**
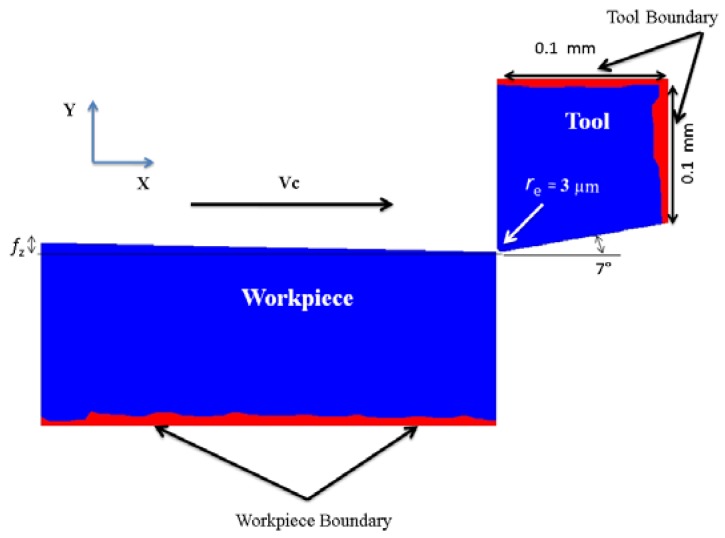
Setup and boundary conditions of the 2D micro end-milling FEM.

Figure 5 shows the setup and the general geometry of the 2D micro end-milling FEM simulation. The workpiece bottom boundary nodes are fixed in the *Y* direction and the tool in both the *X* and *Y* directions. The workpiece moves with a cutting speed or surface velocity, while the tool is stationary. The axial depth of cut is measured in the *Z* direction, perpendicular to the feed and speed directions. The rigid cutting tool was meshed using 670 brick elements. The workpiece was meshed with six-node quadratic triangular elements for a total number of 1566 nodes. The maximum and minimum element sizes for the workpiece were set to 0.1 mm and 0.002 mm, respectively. The maximum element size of the cutter was set at 0.2 mm and the minimum at 0.002 mm. The friction phenomenon at the chip-tool interface was the same as in the 3D FE model, with the same coefficient of friction. A large value of the interface heat transfer coefficient *h_int_* = 10^4^ (N/s mm °C) was selected for these simulations to ensure that they reached a steady state condition.

After running the 2D simulations for all the designed parameter combinations, the maximum forces were extracted from the simulations. The errors between the predicted and the experimental force components (*F_x_* and *F_y_*) were calculated and recorded for all tested combinations. The total error was calculated by (Equation (3)). Each relative error was squared in this equation. Squares were found and summed for each force component and each cutting condition. Subsequently, the sum of the square was divided by *M* × *N* (*M*: number of cutting conditions (4); *N*: number of force components (2)). The square root of this value gives the forces total error, representing the performance of each flow stress model representation. Table 5 shows the total errors for the 16 JC parameter combinations. In the case of the second parameters combination, an error occurred in the simulations preventing us from obtaining results in all cutting conditions.

For these sets of parameters, it was observed that the resultant total error varied between 15.73 and 73.06%. According to these results, the best set of parameters was selected to represent the material flow stress in the following 3D simulations, as presented in Table 6.

(3) $${\mathrm{Error}}_{\mathrm{total}}=\sqrt{\frac{\sum_{j=1}^{M}\sum_{i=1}^{N}[\left(\frac{F_{ji,Ex}-F_{ji,Sim}}{F_{ji,Ex}}{)}^{2}\right]}{N\times M}}$$

## 3. FEM Validation Experimental Procedure

**Figure 6:**
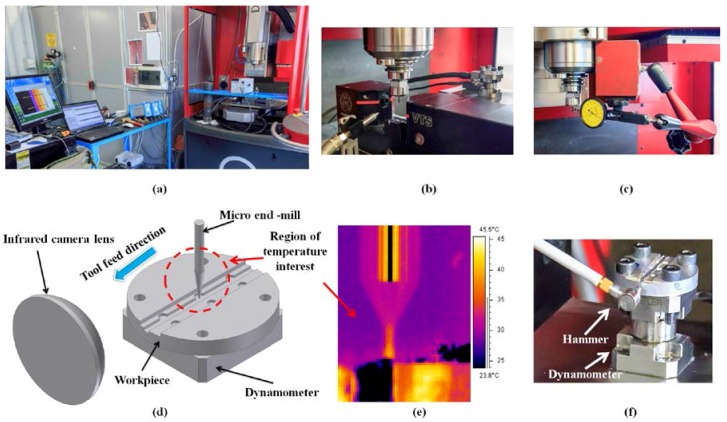
(**a**) Machine tool and experimental setup; (**b**) dynamic run-out measurement; (**c**) static run-out measurement; (**d**) schematic of experimental temperature measurement setup; (**e**) temperature distribution in end-mill; (**f**) dynamometer and force compensation.

In order to verify the accuracy of the developed numerical model, both the predicted cutting forces and temperatures were validated against experiments. The machine tool is one of the critical aspects of micro machining in order to achieve a high precision on machined components and have a stable machining process [44]. Micro milling operations have been performed on a Kern EVO ultra precision 5-axis machining center (nominal positioning tolerance = ±1 μm, precision on the workpiece = ±2 μm). The machine tool and experimental setup are illustrated in Figure 6a.

The cutting tool tips were inspected and measured prior to machining and the only tools with minimal geometrical inaccuracies and defects were selected for the experiments. The run-out of micro end-mills was measured with both static (Figure 6c) and dynamic (Figure 6b) methods. In the static run-out measurements, a dial gauge was used by placing its probe against the tool shank, manually rotating the spindle, and reading the value of run-out as the “total indicator reading” [31]. The measured run-out was 3 ± 1 μm for both tool measurements. The micro mill dynamic run-out was measured by a visual tool setter VTS (Marposs, Bologna, Italy), is equipped with a camera synchronized with the spindle rotation to acquire a set of tool diascopic images at a defined angular step. A dedicated software analyzes these images and calculates the tool diameter, the maximum tool dynamic diameter (or flying circle diameter) *D*_1_, and the radial runout (or *TIR*) *r* [31], corresponding to the difference between the maximum and the minimum tool dynamic radii. The minimum tool dynamic diameter *D*_2_ can be obtained from *D*_1_ and *r* (Figure 4). The experimental setup is shown in Figure 6b. The dynamic measurement was performed at the same rotational speed of the final experiments after the required VTS speed calibration. The tool radial run-out with respect to the spindle axis was 4 ± 0.5 μm and this value was used in the simulations. The measurements were repeated five times in order to guarantee statistical consistency. The standard deviation of the measurement was *XYZ* μm, equivalent to *XY*% of the measured run-out. Micro end-mills were held in HSK32 Kern/Schaublin tool holders and a precision Schaublin collet (type D14, 74-14000) was used to reduce the clamping error and the tool run-out.

Full slot micro end-milling was carried out in dry cutting conditions. The cutting parameters are summarized in Table 7. The cutting conditions were selected according to the literature [45,46], trying to avoid significant rubbing and burr formation due to the feeds per tooth below the cutting edge radius. Four replicates were performed for each cutting condition to consider experimental variability.

### 3.1. Force Measurement

**Figure 7:**
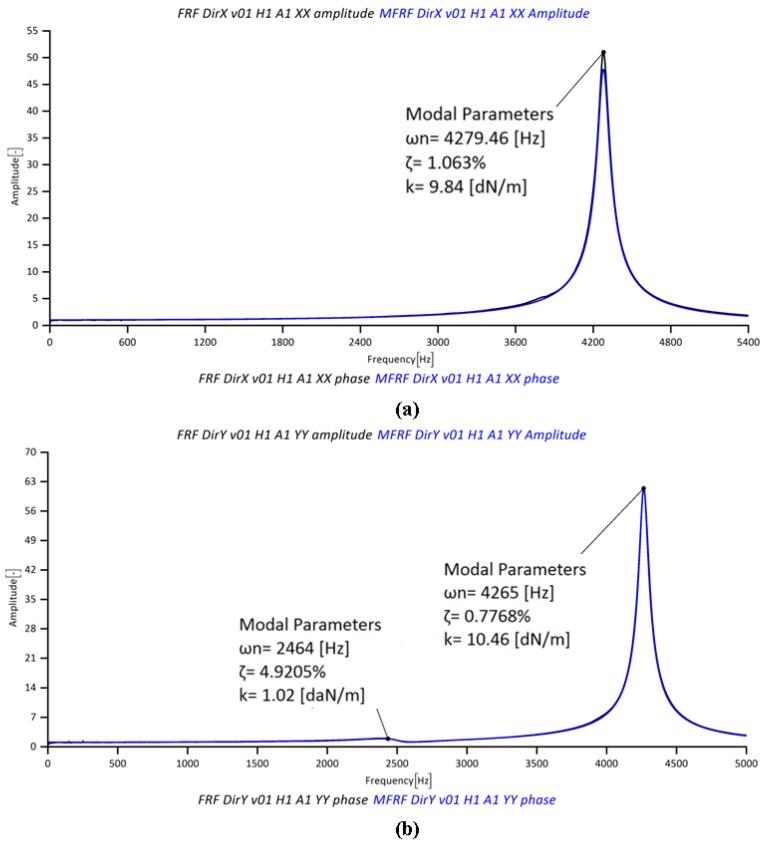
FRFs of the dynamometer in (**a**) *X* and (**b**) *Y* directions.

The cylindrical workpiece (Figure 6f) was fixed on a Kistler 9317B miniature piezoelectric three-axial dynamometer (Figure 6f), which measured the obtained cutting force signals (measuring range: *F_x_*, *F_y_* = ±1000 N, *F_z_* = ±2000 N; linearity error ≤ 0.5% FSO, Full Scale Output) amplified by three Kistler 5015A charge amplifiers. A low-pass filter at 20,000 Hz was directly applied on the charge amplifiers in order to avoid aliasing and a Hanning window was used to reduce leakage. The acquisition time window was one second long for each milling step. The cutting force measurements were affected by vibrations due to the low resonance frequency of the fixturing and force measurement system. In order to accurately measure the micro milling forces, a compensation method was applied to the measured forces [47]. The dynamometer dynamic behavior was identified from the impact tests applied in both the *X* and *Y* directions on the same workpiece material (Figure 6f). Figure 7 shows the result of the impact test used to obtain the frequency response function of the dynamometer in both directions. The same experimental setup and procedure was used for the inverse method experiments for determining the JC parameters (Section “Inverse JC Parameters Estimation with 2D FE Simulations”).

### 3.2. Infrared Temperature Measurements

The infrared ThermaCAM^TM^ SC 3000 Researcher camera (FLIR Systems, Boston, MA, USA) with a waveband in the electromagnetic spectrum (in the range of 8–9 μm) was used for measuring the temperature at the cutting area. The measurement accuracy of the camera was ±1% or ±1 °C (for measurement ranges up to +150 °C) with a spatial resolution (IFOV) of 1.1 mrad. Crisp high-resolution 14-bit images and thermal data were captured and stored at high rates (up to 900 Hz) on high capacity PC. The camera was positioned along the cutting direction (Figure 6d). The temperature measurement was carried out at the tool tip area, as shown in Figure 6e. An infrared camera was calibrated with a set of standard blackbodies at various temperatures. The lenses have their own unique calibration to insure accuracy and the camera will automatically identify the lens attachment and load appropriate data in the radiometric calculations. The emissivity and background correction are variable, from 0.01 to 1.0 [48]. The camera microscope lens was used to acquire images at a 75–99 mm focus range, adjusted on the micro tool with a ±0.05 mm depth of focus. All of the experimental data have been normalized to a room temperature of 21 °C.

## 4. Results and Discussion

### 4.1. Chip Formation

**Figure 8:**
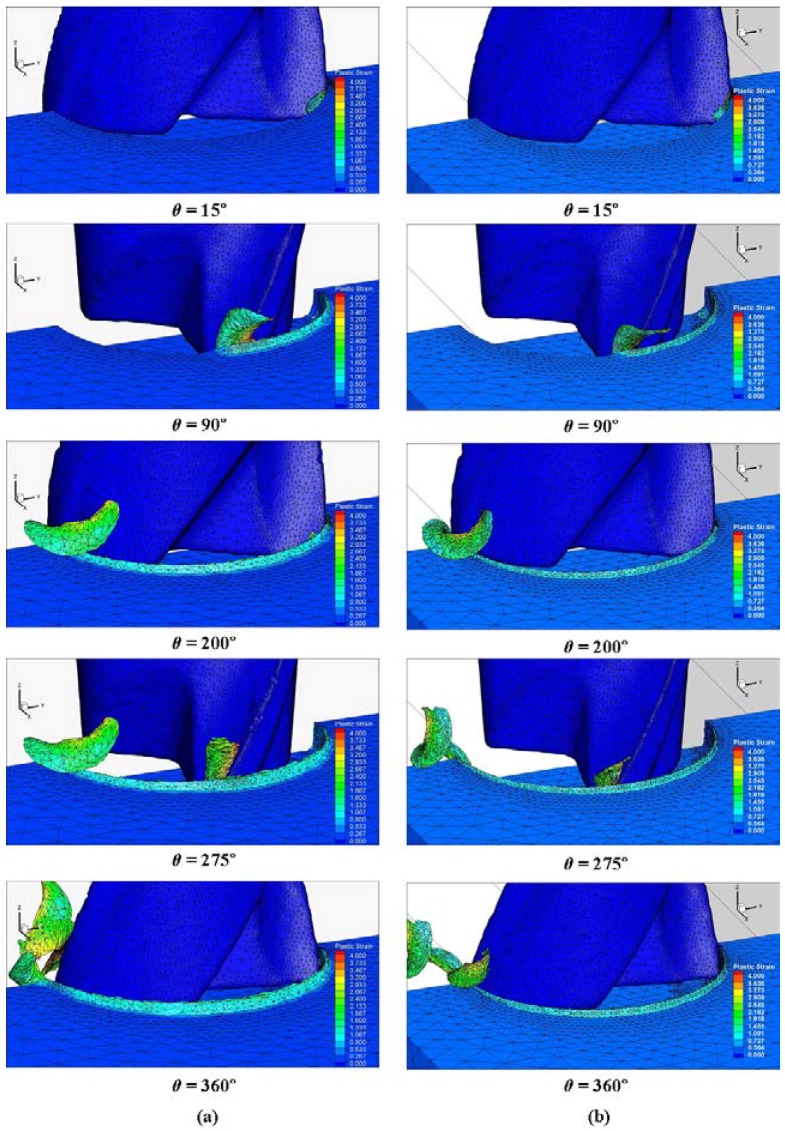
3D chip formation prediction and plastic strain distribution at different angular positions (**a**) Test1 and (**b**) Test2.

**Figure 9:**
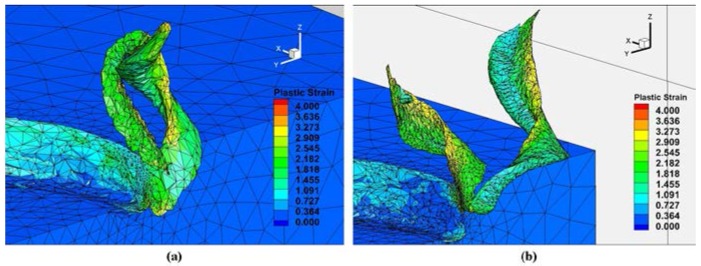
Run-out effect on chip formation in full slot micro milling at (**a**) Test1 and (**b**) Test2.

**Figure 10:**
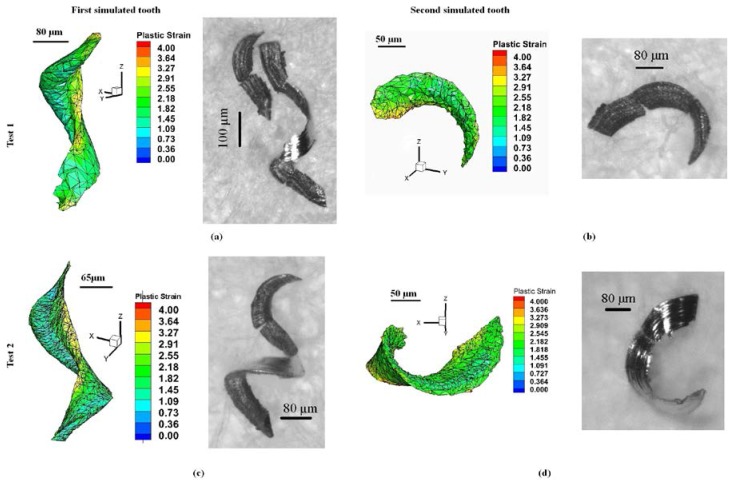
Comparison of predicted and acquired 3D chip shape for full slot micro end-milling of Al6082-T6, (**a**,**b**) Test1 and (**c**,**d**) Test2.

The micro milling full slot operation was simulated for both types of teeth engagements. The calculation time was in the range of 30 ± 3 h, depending on the cutting condition. The different results obtained at the end of the simulations for each cutting condition are presented in this section. Figure 8 shows the chip formation and plastic strain distribution during the micro milling process in two cutting conditions, Test1 (*f_z_* = 8 μm/(tooth·rev)) and Test2 (*f_z_* = 4 μm/(tooth·rev)), for different angular positions of both cutting teeth. The maximum plastic strain occurred in the chip area, mainly near to the end of the cut (about 50 micros from tool tip). As expected, the plastic strain is greater when the feed is higher. The feed effect on the chip formation is also clear since different chip volumes can be observed at the same angular position. Figure 9 shows the chips at the end of the cut (*θ* = 360°). The effects of the tool run-out on the chip shape are visible in Figure 9 and Figure 10. Due to the unequal load, the first and second removed chips present different shapes and sizes.

Figure 10 shows a comparison of the chips obtained from the experiments and simulations. According to the results of the simulations and experiments, the chips formed in the case of lower feeds (Figure 9b) tend to be curlier than in the case of higher feeds (Figure 9b). Figure 10a,c represent the biggest chips, formed by the most engaged tooth because of run-out (the first one) and Figure 10b,d represent the smallest chips, formed by the least engaged tooth (the second one). The experimental chips were collected and correspond to the simulated chip shape as they displayed different sizes and shapes.

### 4.2. Temperature Distribution

**Figure 11:**
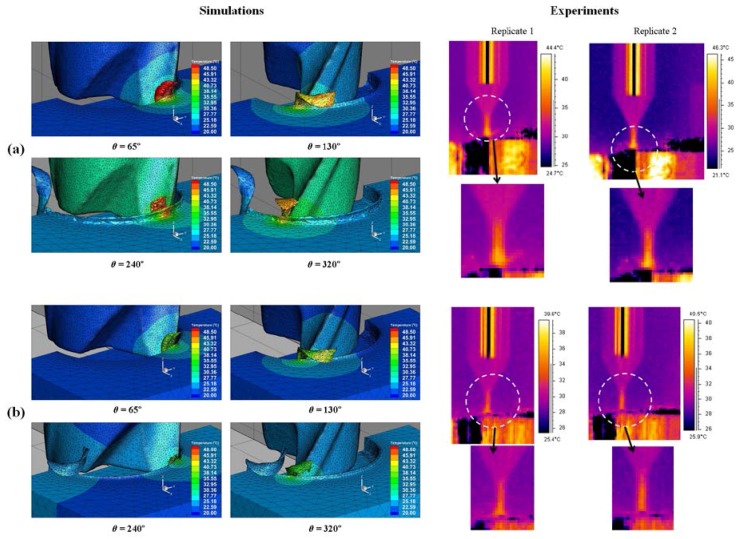
Simulated and experimental temperature distributions in full slot micro end-milling of Al6082-T6 at (**a**) *f_z_* = 8 μm/(tooth·rev) and (**b**) *f_z_* = 4 μm/(tooth·rev).

**Figure 12:**
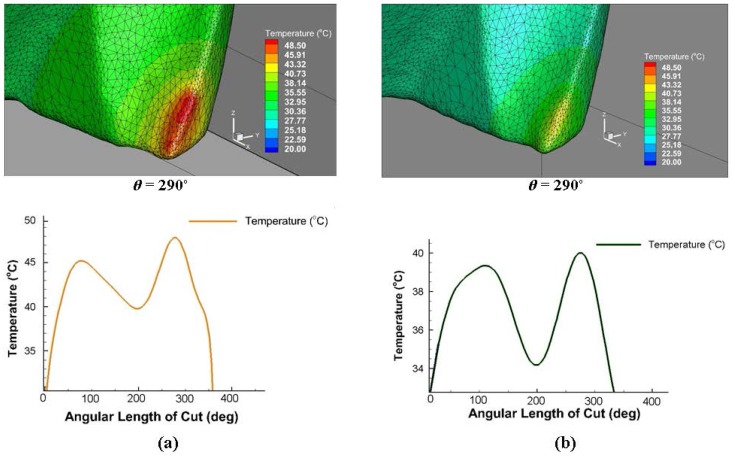
Simulated temperature distribution along the cutting edge for (**a**) Test1 and (**b**) Test2.

**Figure 13:**
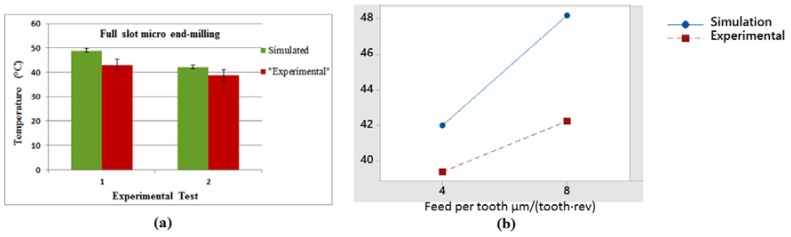
(**a**) Comparison of experimental and simulated maximum temperature distribution and (**b**) interaction plot of temperature distribution

Figure 11 shows the simulated temperature distribution in the cutting area at different angular positions of the tool with the results of two replicates of experimental cutting temperature measurements captured by the thermal camera in the full slot micro end-milling of Al6082-T6. The maximum workpiece temperatures (around 48 °C) in the simulation results can be observed at the chip area. Figure 11b shows the lower feed condition results, where a lower temperature occurs in the chip area and in the tool-workpiece contact zone. Due to the high plastic deformation taking place in the chip formation area, a higher temperature is observed in that region compared to other workpiece positions. Regarding the experimental results, the main region of interest for the temperature measurements was the tool tip, which shows the highest temperature compared to the upper part of the tool. The temperature distributions along the tool cutting edge and the tool-workpiece contact and chip area are shown in Figure 12. The variation of the highest temperature pick in different engagements toward the end of cut is presented for both cutting conditions. The tool maximum temperature region is at the corner radius and rake face of the micro cutter. The graphs of cutting tool temperature distributions (Figure 12) indicate dissimilar temperature drops after the first tooth engagement at *θ* = 200° and at the end of the cut caused by the feed rate variation at different cutting conditions. It can be seen that more realistic results of the temperature distributions were obtained to comprehend the effect of different cutting conditions in both the chip and cutting edge with the 3D FEM, as it is nearly impossible to monitor each cutting edge in physical experiments. Figure 13a shows the maximum temperature distribution comparison from the experimental and simulation results in the cutting area and Figure 13b presents the interaction plot from the design of experiments (DOE) analysis. The results indicate that cutting parameters influence the temperature distribution, and a lower temperature was observed in a lower cutting condition. The interaction plot shows that the simulation results were overestimated by between 7% and 12.5% in comparison with the experiments; however, a similar trend was observed with the experiments. The temperature deviation was in the range of 3–6 °C.

### 4.3. Cutting Forces Predictions

**Figure 14:**
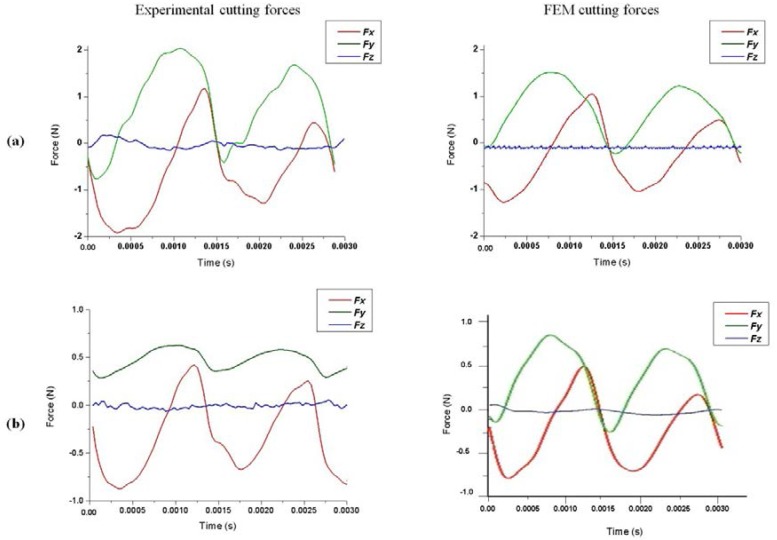
Experimental and FEM cutting force curves for (**a**) Test1 and (**b**) Test2.

It was noted that all the simulated forces were affected by a relevant amount of numerical noise distributed at an extremely high frequency range, much beyond the range of real cutting force contributions. A fifth-order Butterworth low-pass filter was therefore applied to the simulated signals with a cut-off frequency equal to the bandwidth achievable by the dynamometer installed on the machine, which is around 4000 Hz (after the application of the acquired force compensation [41]), in order to obtain simulated force signals comparable with the experiments. The comparisons were performed between results obtained by the finite element model and the physical experiments. Figure 14 shows the cutting force components *F_x_*, *F_y_*, and *F_z_* obtained for both teeth engagements, considering the effect of the run-out in the full slot micro end-milling process. The comparison between the experiments and simulation reveals globally similar trends for *F_x_*, and *F_y_* components in terms of the curve shapes. However, some discrepancies in the magnitude of forces were observed, probably due to aluminum particles sticking to the tool (build up edge) that affect the tool geometry and the tool dynamics. The effect of the run-out phenomenon is visible in the cutting force results as the first tooth shows higher values of the *F_x_* and *F_y_* components due to the larger material removal. The *F_z_* component was almost constant during the cutting operation in both the experimental and simulation results.

## 5. Conclusions

A 3D FEM was developed in this work for studying the micro end-milling process on aluminum alloy 6082-T6. The advantages of 3D FE simulations were used with additional analyses on the effect of tool run-out. The model was applied to investigate the chip formation, cutting tool temperature distribution, and cutting forces in full slot milling. The simulation results were compared against a series of experiments. Some conclusive findings of this study are outlined in the following:

- The constants of the Al6082-T6 Johnson-Cook material constitutive model were obtained through an inverse method based on an experimental analysis on the cutting forces. Different combinations of parameters were employed to determine proper constants by comparing the experimental and simulation results.
- The chip formation was observed in realistic simulations thanks to the 3D FEM approach. An unequal chip load caused by run-out was obtained in both the experiments and simulations, together with different chip shapes for the two teeth engagements.
- Higher temperatures were observed in high feed rate conditions at the tool cutting edge radius and the maximum temperature distribution was different along the cutting edge at various angular positions of the tool. The highest temperature distribution was on the chips and tool cutting edge radius area. Experimental temperature distributions were mainly observable on the tool area and were in line with simulation results.
- The *F_x_*, *F_y_*, and *F_z_* cutting force components were obtained as FEM predictions and experimental results for the two teeth, pointing out their different cutting action due to run-out.

The comparison between 3D simulations and experiments confirms that the model was capable of predicting the main features of the complex micro end-milling process on 6082-T6 aluminum alloy.

## Figures and Tables

**Table 1:** Nominal and actual tool characteristics. (*) the interval refers to the standard deviation/the uncertainty of the measurement.

Title	Nominal Dimensions	Actual Dimensions
Tool manufacturer	Dormer	
Code	S150.05	
Tool material	Carbide	
Surface treatment	TiAlN-X	
Flute number	2	
Diameter	500 μm	492.0 ± 2 μm (* 1.47)
Cutting edge radius (*r_e_*)	-	3.0 ± 1 μm (* 0.71)
Helix angle	30°	27.26°
Rake angle	0°	0°
Relief angle	8°	7°
Corner radius (*r*_ε_)	20 μm	22 μm

**Table 2:** Experimental and 2D simulated cutting conditions.

Cutting Conditions	Tool Diameter	Cutting Speed	Radial Depth of Cut (*a_e_*)	Axial Depth of Cut (*a_p_*)	Feed Per Tooth	*F_x_*	*F_y_*
	(mm)	(m/min)	(mm)	(mm)	(μm/(Tooth·Rev))	(N)	(N)
1	0.5	28	0.125	0.05	4	0.7	0.3
2	0.5	28	0.25	0.05	4	0.93	0.8
3	0.5	28	0.125	0.1	4	1.3	0.64
4	0.5	28	0.25	0.1	4	1.63	1.2

**Table 3:** Johnson-Cook constants for Al6082-T6.

Level	*A* (MPa)	*B* (MPa)	*C*	*m*	*n*	
1	428.5	327.7	0.00747	1.31	1.008	[39]
2	214.25	163.85	0.003735	1.31	0.504	

**Table 4:** Material properties for Al6082-T6 [43].

Property	Unit	Value
Young’s modulus, *E*	(GPa)	70
Poisson ratio, *v*	-	0.33
Density, *ρ*	(g/cm^2^)	2.70
Thermal conductivity, *K*	(W/m·K)	180
Specific heat, *C*p	(J/Kg·°C)	700
Thermal expansion coefficient	-	24 × 10^−6^
Melting temperature, *T_melt_*	(°C)	582

**Table 5:** Simulated force results and comparison with experiments.

No	Coded Variable				Cutting Conditions								
	*A*	*B*	*n*	*C*	1		2		3		4		
					*F_x_*	*F_y_*	*F_x_*	*F_y_*	*F_x_*	*F_y_*	*F_x_*	*F_y_*	Total Error
	(MPa)	(MPa)			(N)	(N)	(N)	(N)	(N)	(N)	(N)	(N)	(%)
**1**	428.5	327.7	1.008	0.003735	1.4	0.33	1.77	0.4	2.4	0.7	3.2	0.82	65.56
**2**	214.25	163.85	0.504	0.00747	0.06	0.04	0.06	0.04	0.1	0.14	0.09	0.13	Error
**3**	214.25	327.7	1.008	0.003735	0.3	0.15	1.4	0.3	0.6	0.3	2.7	0.5	57.81
**4**	214.25	327.7	1.008	0.00747	1	0.24	1.2	0.58	0.83	0.42	2.1	0.75	31.13
**5**	428.5	327.7	1.008	0.00747	1.3	0.32	1.7	0.4	2.6	0.7	3.8	0.8	73.06
**6**	428.5	327.7	0.504	0.003735	0.82	0.43	1.1	0.57	1.7	0.88	2.4	1.1	24.7
**7**	428.5	163.85	0.504	0.00747	0.54	0.35	0.72	0.65	1.1	0.6	0.83	0.61	30.88
**8**	428.5	163.85	0.504	0.003735	0.55	0.24	0.86	0.38	0.98	0.65	1.5	0.8	28.38
**9**	428.5	163.85	1.008	0.00747	0.97	0.33	1.33	0.4	1.95	0.7	2.6	0.8	39.71
**10**	214.25	163.85	1.008	0.003735	0.65	0.16	0.08	0.14	1.4	0.3	0.45	0.2	64.24
**11**	214.25	163.85	1.008	0.00747	0.82	0.21	0.17	0.09	1.5	0.35	0.35	0.18	62.57
**12**	214.25	327.7	0.504	0.00747	0.83	0.3	0.98	0.56	1.48	0.63	1.78	0.89	15.73
**13**	214.25	163.85	0.504	0.003735	0.54	0.24	0.7	0.3	1.03	0.46	1.4	0.6	35.93
**14**	428.5	327.7	0.504	0.00747	1	0.47	1.4	0.6	2	1	2.75	1.2	47.68
**15**	428.5	163.85	1.008	0.003735	0.3	0.19	1.23	0.34	1.9	0.65	2.5	0.73	45.51
**16**	214.25	327.7	0.504	0.003735	0.85	0.27	0.65	0.54	1.6	0.53	0.92	0.83	27.87

**Error:** with the following material combination constants simulations did not finished to the end.

**Table 6:** Best set of JC Al6082-T6 flow stress model constants used for 3D simulations.

*A* (MPa)	*B* (MPa)	*C*	*m*	*n*	*T_m_* (°C)	*T_a_* (°C)
214.25	327.7	0.00747	1.31	0.504	582	21

**Table 7:** Experimental tests conditions.

Cutting Parameters	Symbol	Test1	Test2
Axial depth of cut (mm)	*a_p_*	0.05	0.05
Radial depth of cut (mm)	*a_e_*	0.5	0.5
Feed per tooth (μm/(tooth·rev))	*f_z_*	8	4
Cutting speed (m/min)	*v_c_*	31.4	31.4
Spindle speed (rpm)	*n*	20,000	20,000
Approach		Full slot	
